# Facial Expressions of Emotions During Pharmacological and Exercise Stress Testing: the Role of Myocardial Ischemia and Cardiac Symptoms

**DOI:** 10.1007/s12529-021-09963-3

**Published:** 2021-02-23

**Authors:** Maria T. Bekendam, Willem J. Kop, Ilse A. C. Vermeltfoort, Jos W. Widdershoven, Paula M. C. Mommersteeg

**Affiliations:** 1 Center of Research on Psychology in Somatic Diseases (CoRPS), Department of Medical and Clinical Psychology, Tilburg University, Warandelaan 2, P.O. Box 90153, 5000 LE Tilburg, The Netherlands; 2 Department of Nuclear Medicine, Institute Verbeeten, Tilburg, The Netherlands; 3 Department of Cardiology, Elizabeth-TweeSteden Hospital, Tilburg, The Netherlands

**Keywords:** Cardiac stress testing, Cardiac symptoms, Emotions, Face reader software, Myocardial ischemia, Myocardial perfusion imaging

## Abstract

**Background:**

Negative emotions have been linked to ischemic heart disease, but existing research typically involves self-report methods and little is known about non-verbal facial emotion expression. The role of ischemia and anginal symptoms in emotion expression was examined.

**Methods:**

Patients undergoing cardiac stress testing (CST) using bicycle exercise or adenosine with myocardial perfusion imaging were included (*N* = 256, mean age 66.8 ± 8.7 year., 43% women). Video images and emotion expression (sadness, anxiety, anger, and happiness) were analyzed at baseline, initial CST , maximal CST, recovery. Nuclear images were evaluated using SPECT.

**Results:**

Ischemia (*N* = 89; 35%) was associated with higher levels of sadness (*p* = .017, *d* = 0.34) and lower happiness (*p* = .015, *d* = 0.30). During recovery, patients with both ischemia and anginal symptoms had the highest sadness expression (*F* (3,254) = 3.67, *p* = .013, eta^2^ = 0.042) and the lowest happiness expression (*F* (3, 254) = 4.19, *p* = .006, eta^2^ = .048).

**Conclusion:**

Sadness and reduced happiness were more common in patients with ischemia. Also, anginal symptoms were associated with more negative emotions.

## Introduction

Negative psychological factors such as depression, anxiety, and anger are common in patients with ischemic heart disease [1–3]. These psychological factors are known to adversely affect clinical outcomes in cardiac patients [1, 2, 4]. In addition, positive psychological factors are increasingly recognized as potentially protective factors for health in general [5, 6] and incident and recurrent ischemic heart disease in particular [7]. A bi-directional association may exist between inducibility of myocardial ischemia and adverse (high negative or low positive) psychological factors [8]. One well-established pathway is that negative emotions can elicit myocardial ischemia during daily life activities and during laboratory-based mental stress testing [2, 9–11]. However, it is also possible that another pathway is important in which myocardial ischemia results in a lower threshold for the experience and expression of negative emotions, but this direction of the association is much less well investigated. Exertion-related chest pain and other angina equivalents are the core clinical manifestation of chronic ischemic heart disease. However, the relationship between transient myocardial ischemia and anginal complaints is complex, with inter- and intra-individual variability, including individuals with silent ischemia, symptomatic ischemia, and cardiac complaints without documented inducible ischemia [12]. Evidence suggests that approximately 85% of patients with ischemic heart disease display silent ischemia during daily life and one-third to half of the patients report anginal complaints during diagnostic testing in the clinic. In addition, during cardiac stress testing, only one-third of the patients reporting anginal complaints also display inducible myocardial ischemia, providing further support for the uncoupling of ischemia and chest pain [13]. The pathway linking myocardial ischemia to a lower threshold of negative emotions is particularly relevant for patients who have ischemia-related cardiac symptoms such as chest pain, dyspnea, and other angina equivalents. The present study aims to disentangle the role of induced myocardial ischemia versus reported cardiac symptoms as related to momentary responses of expressed emotions in patients undergoing cardiac stress testing (CST) for the inducibility of myocardial ischemia.

To date, studies on emotions and myocardial ischemia have focused on measuring emotions based on self-report. This approach, however, may only partially reveal dynamic information about cardiac symptoms and emotions [14]. Emotions and cardiac symptom reports are inter-related [15, 16], but part of this association may reflect negative self-report tendencies as a common underlying factor (e.g., neuroticism and negative affectivity) [17]. Evidence indicates that patients with inducibility of myocardial ischemia during CST combined with anginal symptoms have elevated levels of anxiety and depression [18] and that factors such as the severity of ischemia and biological factors related to pain perception were not associated with anginal complaints during CST [19]. However, patients without ischemia were not included in that study, precluding inferences about patients with suspect ischemic heart disease who have anginal complaints but no evidence for inducible myocardial ischemia. It is therefore important to use methods that do not solely rely on self-report, such as measures of facial emotional expression derived from digital video images. Several studies have focused on expressions of emotions that do not depend on self-report, with a particular emphasis on facial expressions of emotions [20, 21]. Systematic assessment tools such as the Facial Action Coding System (FACS) have been successfully employed in several clinical and research settings [22, 23]. Previous studies show that facial expressions of emotions have distinct features (action units in dynamic facial expression) that differ from facial expressions of pain [24]. But there is also overlap between the characteristic expressions of pain and negative emotions [25]. It is therefore important to take cardiac symptoms into account when examining the association between ischemia with expression of emotions.

Building on advances in automated facial coding, the current study measures facial expressions of emotions during CST and examines associations with the presence of ischemia and anginal symptoms. Previous research on emotions in patients with ischemic heart disease mainly found depression, anxiety, anger, and reduced levels of positive affect to be involved [1, 2]. Earlier studies have examined these negative emotions as predictors or triggers of myocardial ischemia [26–28], but little is known about the effects of ischemia and anginal symptoms with CST on negative emotions. It is hypothesized that: (1) Patients with ischemia show significantly more facial expressions of negative emotions (sadness, anxiety, anger) and less positive emotions (happiness) during CST than patients without ischemia. (2) The facial expressions of negative emotions (sadness, anxiety, anger) and (lower) happiness are significantly related to chest pain and other anginal symptoms reported during CST. Additionally, we will also explore whether ischemia and anginal symptoms display an additive effect in their association with emotional expressions during CST, and whether there is an interaction effect between ischemia and anginal symptoms. Unique to this investigation is the reliance on analysis of digitized video recordings of emotional expression during CST that may complement self-report data in patients undergoing CST.

## Method

### Patients and Procedure

The Heart Inside Out (THIO) study is a prospective, single center study examining the relationships between psychosocial factors, digitally assessed emotion expression, and symptoms during CST in patients undergoing myocardial perfusion imaging. Patients who were referred for myocardial perfusion imaging by their cardiologist were eligible for this study. Referral for CST was based on clinical factors (e.g., cardiac symptoms suggestive of coronary heart disease, high risk profile based on cardiovascular risk factors, or routine clinical follow-up related to coronary revascularization interventions).The project was conducted at Institute Verbeeten, Tilburg, the Netherlands between January 2017 and December 2018.

Inclusion criteria were (1) referral for CST with myocardial perfusion imaging, (2) ability to fill out questionnaires, and sufficient knowledge of the Dutch language. There were no exclusion criteria.

Prior to CST, patients were informed about the aim and scope of the study via e-mail, which they received along with their invitation for the clinically indicated CST. Upon arrival on the day when baseline (resting) perfusion images were obtained, patients were informed in detail about the study by a member of the research staff and provided informed consent. All patients underwent the protocol as described below (see also the flow chart presented in Figure S1) with myocardial perfusion images obtained at rest and following CST. CST entails either bicycle exercise stress testing (physical exertion) or pharmacological adenosine stress testing (in sitting position with minimal resistance bicycling to distribute the pharmacological agent, or lying down if minimal exertion is not possible); these protocols will be referred to as CST in the remainder of this paper. During CST, patients’ facial expressions were video-recorded using a webcam (Logitech C920-HD Pro) attached to the stationary exercise bicycle (GE Healthcare, Ergometer ebike comfort 162202, Freiburg, Germany). Sociodemographic and psychosocial data were collected before, during, and after CST (see below for details). The study was approved by the local Medical Ethics Committee (METC Brabant, Protocol number: NL56707.028.16).

Of the 297 patients enrolled in the study, valid digital data for emotional expression were available for 256 (86%) patients. The reason for non-inclusion in the present analyses were attrition (*N* = 1), poor quality of the video recording (*N* = 6), or scheduling problems (e.g., unavailability of research staff because CST rescheduling related to patients not adhering to medication/caffeine guidelines; *N* = 34).

### Design

This is a cross-sectional study of patients undergoing CST with myocardial perfusion imaging for the detection of inducibility of myocardial ischemia. To address the research question of this study, four groups were compared based on the cardiac responses (ischemia vs. no ischemia during CST) and symptomatic responses (anginal symptoms during CST): This approach enabled the comparison of four groups (i.e., (1) no ischemia-no angina (reference group), (2) no ischemia-angina (non-ischemic complaints), (3) ischemia-no angina (silent ischemia), and (4) ischemia-angina (symptomatic ischemia)) with regard to emotion expression. We will examine main effects for inducibility of ischemia (present vs. absent), the main effect of symptoms during CST (presence vs. absence of anginal complaints during CST), and the interaction between these two factors as related to emotion expression (assessed by FaceReader analyses or self-report).

### Cardiac Rest and Stress Testing Protocol

The timing of the myocardial perfusion imaging protocol and study-related assessments are displayed in Figure S1. The protocol consists of two separate imaging days. The first day (perfusion imaging at rest) consisted of injection of the tracer (Technetium myoview tetrofosmin; 99mTc; dosage: 370 MBq), and a rest period of 45 min followed by (rest) myocardial perfusion imaging. At the second day (perfusion imaging after CST), patients performed CST, either by physical exertion (i.e., bicycling to maximum exertion using the modified Bruce protocol), or pharmacologically by intravenous adenosine injection (140 mcg/kg/minute for 5 min). As per standard clinical protocol, the pharmacological adenosine CST also involved mild-intensity cycling, aimed to distribute the adenosine in the blood circulation, limit adenosine side-effects, and reduce extracardiac activity [29]. If cycling was not possible, patients were asked to lie down on a bed while adenosine was administered (*N* = 45). On average, 2 or 3 days were scheduled between the rest and CST day.

During CST with physical exercise, the tracer was injected when the patient reached 85% of his or her maximum heartrate (0.85∗(220-patientage). For CST with adenosine, the tracer was injected at 2 min after adenosine administration. Myocardial perfusion images were obtained at 45 min post injection on both the resting day and the day of CST. Perfusion images were inspected by qualified staff before interpretation by the nuclear physicians.

### Myocardial Perfusion Imaging

Patients underwent single-photon emission computed tomography myocardial perfusion imaging scans on the rest day and CST day. The interpretation of perfusion images was performed by an experienced nuclear physician, and each interpretation was validated by a second nuclear physician. Summed difference scores (SDS) between stress and resting images, indicating the magnitude of inducible perfusion abnormalities, were calculated from 17-segment cardiac images. Visual interpretation of the myocardial perfusion imaging was assessed on both semiquantitative (SDS) and visual analysis as is recommended by the American Society of Nuclear Cardiology (ASNC) [30]. The myocardial perfusion imaging results (based on SDS and overall difference in perfusion between rest and CST) was used as evidence of presence/absence of myocardial ischemia: the primary index of myocardial ischemia used in the statistical analyses. In addition to SDS and presence/absence of ischemia, the left ventricular ejection fraction (LVEF) was calculated during rest and CST.

### Video-Recordings of Facial Emotion Expression During CST

Video-recordings were made on the day of CST using a webcam attached to the exercise-bicycle. The recordings were divided into four consecutive time blocks, “baseline,” “start CST,” “maximal CST,” and “recovery,” as depicted in Figure S1.

- 1. Baseline
  After taking a seat on the exercise-bicycle (physical exertion or adenosine), or laying down (adenosine only), the video recordings started with “baseline,” approximately 1 min before cardiac stress testing actually began, and served as calibration and baseline measure of facial expression. Patients were asked to look into the camera with a neutral expression for 5 s to obtain a calibration image for that particular position.

- 2. Start CST
  Start CST commenced when patients were instructed to start cycling on the exercise-bicycle (physical exertion) and adenosine injection coincided with the start of mild cycling. Depending on whether CST was performed by physical exertion (cycling up till 85% of maximum heart rate) or with adenosine injection, the duration of the “start CST” time block lasted several minutes (physical exertion) or 2 min (adenosine), respectively. Analyses of the video images were adjusted for the duration of the recordings by examining the percentage of time of each expressed emotion (see below for details).

- 3. Maximal CST
  The maximal CST video time block started immediately after injection of the tracer. After tracer injection, the patient (both with physical exertion and adenosine) was instructed to continue cycling for 1 min, which defines the maximal CST time block.

- 4. Recovery
  Patients were asked to stop mild cycling at 1 min post tracer injection (adenosine protocol), or to slowly come to a stop at 1 min post tracer injection (physical exertion). Recovery video images were obtained during this slowing down and stopping phase (approximately 1 min), while patients remained in the same position as during CST.

### Digital Analysis of Facial Expression

The software package FaceReader 7.0 [31] was used to analyze the video-recordings for the abovementioned four time blocks obtained during CST. Analysis took place at the GO-LAB (“Gedragsfysiologisch Onderzoekslaboratorium”; Behavioral-physiological research laboratory) of Tilburg University. The FaceReader software finds a patient’s face and creates a 3D Active Appearance Model of the face [32]. Additionally, FaceReader uses deep artificial network analysis to recognize patterns in the face [33]. This Active Appearance Model combined with pattern recognition is used to compute scores of intensity of facial expressions on a continuous scale from 0 to 1, yielding the percentage (0–100% in a specific time block) of displayed basic or universal emotions sadness, anxiety, anger, and happiness, the target emotions of the present study, as well as surprise and disgust (not included in this study). In each video, 15 frames per second are analyzed [34], and a mean percentage of each emotion during each consecutive CST event (baseline, start CST, maximal CST, recovery) was reported. Also, the number of actual analyzed frames for each patient was recorded for the entire video, as well as for each CST time block separately. Analysis of the present study was based on all available frames obtained during the four time blocks. The mean number (± SD) of recorded frames per time block were baseline 2367.8 ± 2013, start CST 2436.9 ± 1781.0 maximum CST 1818.8 ± 2250.5, and recovery 1107.0 ± 574.7.

Before each individual video-recording was analyzed, the patient’s face was calibrated over a consecutive two second period (30 frames out of the 5-s calibration phase) in which FaceReader automatically corrects for person-specific features that could result in biases towards a certain facial expression.

### Cardiac Symptoms and Self-Reported Anxiety During CST

During CST patients were asked: (a) whether they experienced recognizable anginal symptoms (present/absent) similar to their “typical” cardiac symptoms and (b) to rate the severity of specific symptoms during the CST on a scale from 1 to 10, with the absence of symptoms rated as zero. These symptoms included chest pain, shortness of breath, dizziness, nausea, fatigue, hot flushes, and other (less common with CST) symptoms, such as sore legs, dry throat, or headache. Both the presence/absence and continuous scores of the symptoms are reported.

At baseline and during CST (at 2-min intervals), patients were also asked if they felt anxious or tense and to rate that feeling on a scale from 1 to 10; as with cardiac symptoms, the scale was coded zero if anxiety/tension was absent.

### Cardiovascular Risk Factors, Cardiac History, and Demographic Background Variables

Cardiovascular risk factors were retrieved from hospital records, including hypertension, hypercholesterolemia, and familial risk (first degree family members with cardiac disease, younger than 60 years). Cardiac history included previous coronary angiography (CAG), and having undergone multiple previous CAG’s. Medication use, including anticoagulants, statins, hypertension medication, and nitrates, was also recorded.

Patients completed a questionnaire to obtain information about sociodemographic factors (age, sex, educational level (college education or higher versus lower), living with partner or not, and lifestyle-associated cardiovascular risk factors such as current smoking (yes/no), alcohol use (yes/no), and physical activity (daily 30 min moderate exertion: yes/no).

### Statistical Analyses

Data are presented as mean ± standard deviation (SD) or frequency (N) and percentages. The primary outcome measures are the facial expressions of emotions sadness, anxiety, anger, and happiness.

Comparisons between patients with versus without myocardial ischemia were examined using independent samples *t* tests for continuous variables and chi^2^ tests for categorical variables. Continuous variables with skewed distributions (cardiac symptoms reported during CST and anxious/tense) were compared using Mann-Whitney *U* tests for group differences.

The facial expression of emotions during the CST time blocks (start CST, maximum CST, recovery) were compared with the baseline levels using repeated measures analysis of variance (ANOVA) and paired samples *t* tests. To examine the role of ischemia in facial expressions of emotions during CST, mixed model repeated measures ANOVAs were used to examine differences between facial emotion expressions during the four CST time blocks, with CST time blocks as within-subjects factor, and inducibility of ischemia (presence or absence of ischemia) as between-subjects factor, using Greenhouse-Geiser correction if assumptions of sphericity were not met [35].

For the associations between cardiac symptoms during CST with facial emotional expression, two sets of analyses were performed: (a) Associations between the occurrences of patients’ “typical” anginal symptoms with emotional expression were examined using *t* tests and (b) associations with the severity of symptoms during CST (rated on the 10-point scale) were examined using Kendall’s tau-*b* correlation analyses (*τ*_*b*_). For these analyses, symptoms reported during maximum CST were examined because they are expected to be highest during that time block of the protocol.

To explore whether ischemia and presence versus absence of anginal symptoms displayed an additive effect on facial expressions of emotions, one-way ANOVA analyses were performed in which four groups were compared: no ischemia-no angina (reference group), no ischemia-angina (non-ischemic complaints), ischemia-no angina (silent ischemia), and ischemia-angina (symptomatic ischemia). Additionally, to examine potential synergy between ischemia and CST-induced angina, a two-way repeated measures ANOVA was performed to examine the interaction between ischemia and anginal symptoms on facial expressions of emotions.

Explorative post hoc analyses examined whether the facial expressions of emotions differed by the type of CST (bicycling exercise versus adenosine), and patients’ sex using independent sample t-tests, as both factors may play a role in the association between ischemia with emotions [36, 37]. Assumptions were checked prior to conducting the statistical analyses and two-sided *p* values are reported with a *p* < 0.05 considered as statistically significant. Effect sizes are reported as Cohen’s *d* for *t* tests and partial eta^2^ for ANOVAs. Statistical analyses were performed using the Statistical Package for the Social Sciences (SPSS) version 24 for Windows (SPSS Inc., Chicago, IL).

## Results

Descriptive statistics of demographic variables, cardiovascular risk factors, and cardiac history stratified for ischemia are presented in Table 1. Myocardial ischemia was present in 89 patients (35%). The mean age of patients with myocardial ischemia was 66.4 ± 8.2 years vs. 67.7 ± 9.6 for patients without myocardial ischemia (*t*(254) = 1.10, *p* = 0.113). Patients with myocardial ischemia were more likely to have undergone a previous CAG, or multiple CAG’s (*X*^2^ = 13.07, *p* =  ≤ 0.001; *X*^2^ = 7.38, *p* = 0.007), had a previous history of percutaneous coronary intervention (PCI), and were also more likely to use anticoagulants, nitrates, and diabetes-related medications (see Table 1).

**Table 1:** Characteristics of patients stratified by the presence or absence of inducible myocardial ischemia

		No myocardial ischemia (*n* = 167)	Myocardial ischemia (*n* = 89)	Test-value
	N	Mean ± SD or N (%)	Mean ± SD or N (%)	t or X^2^
Sociodemographic factors				
Age (years)	256	66.37 ± 8.16	67.67 ± 9.55	1.10
Women	256	78 (47%)	32 (36%)	2.74
College education or higher	252	113 (68%)	54 (63%)	.707
Living with partner	254	136 (81%)	75 (86%)	.925
Cardiovascular risk factors				
Smoking (current)	253	26 (16%)	12 (14%)	.156
Physical activity (inactive)	248	27 (17%)	8 (10%)	2.21
Alcohol use (none)	250	53 (33%)	37 (43%)	2.47
BMI (kg/m^2^)	245	28.12 ± 5.60	28.68 ± 6.01	.712
Obese (BMI ≥ 30 kg/m^2^)	245	45 (28%)	28 (33%)	.616
Hypertension	227	69 (47%)	41 (51%)	.386
Hypercholesterolemia	218	68 (48%)	36 (47%)	.043
Family history of CVD	250	96 (58%)	51 (60%)	.077
Cardiac history				
Previous CAG	256	73 (44%)	60 (67%)	13.07**
Previous multiple CAG (≥ 1)	256	25 (15%)	26 (29%)	7.38*
PCI	254	37 (22%)	33 (38%)	7.13**
CABG	254	21 (13%)	16 (18%)	1.56
Previous MI	254	19 (11%)	11 (13%)	.088
Medication				
Anticoagulants	256	114 (68%)	75 (84%)	7.70*
Βeta-blocker	256	85 (51%)	49 (55%)	.402
Statins	256	97 (58%)	61 (69%)	2.69
ACE/ARB-inhibitors	256	81 (49%)	47 (53%)	.431
Nitrates	256	46 (28%)	36 (40%)	4.44*
Diabetes-related	256	24 (14%)	25 (28%)	7.06*
Thyroid medication	256	16 (10%)	4 (5%)	2.09
Antidepressants	256	13 (8%)	7 (8%)	.001

*BMI* body mass index, *CVD* cardiovascular disease, *CAG* coronary angiography, *CABG* coronary artery bypass graft surgery, *MI* myocardial infarction, *PCI* percutaneous coronary intervention

^*^p < .05; ^**^p < .01 for comparison of patients with versus without inducibility of myocardial ischemia

Details regarding CST are presented in Table 2, stratified for the presence or absence of myocardial ischemia. No significant differences in LVEF were found between patients with versus without ischemia. Of the 256 patients, 177 (69%) were examined using the adenosine protocol (132 while sitting during minimal exertion and 45 while lying down) and 79 (31%) performed the exercise bicycling modified Bruce protocol. The frequency of ischemia did not differ between the two protocols (Table 2).

**Table 2:** CST and symptoms stratified for myocardial ischemia

		No myocardial ischemia (*n* = 167)	Myocardial ischemia (*n* = 89)	Test-value
Cardiac stress testing (CST)	N	Mean ± SD or N (%)	Mean ± SD or N (%)	t/Χ^2^/U
Summed Difference Score (SDS)	256	0.36 ± 0.91	4.19 ± 3.70	−9.62***
Adenosine cardiac stress testing (versus cycling)	256	111 (67%)	66 (74%)	1.61
LVEF (% at rest)	235	60.25 ± 13.74	58.13 ± 11.74	1.24
LVEF (% at exertion)	234	59.14 ± 13.59	56.07 ± 12.16	*1.77*
Anginal symptoms present during CST	256	57 (34%)	28 (32%)	.187
Symptoms reported^a^	249	147 (91%)	75 (86%)	1.20
Chest pain	249	2.23 ± 3.20; 59 (36%)	2.16 ± 3.01; 35 (40%)	7.11
Shortness of breath	249	2.86 ± 3.12; 88 (54%)	2.31 ± 3.09; 38 (44%)	6.33
Dizziness	249	1.51 ± 2.50; 54 (33%)	1.96 ± 2.89; 35 (40%)	7.61
Nausea	249	0.75 ± 2.02; 25 (15%)	0.77 ± 2.18; 12 (14%)	6.96
Fatigue	249	2.24 ± 3.41; 53 (33%)	1.85 ± 2.99; 28 (32%)	6.79
Flushing	249	1.94 ± 2.74; 74 (46%)	1.25 ± 2.06; 31 (36%)	*6.17*
Other symptoms	249	1.74 ± 2.81; 55 (34%)	1.91 ± 2.77; 32 (37%)	7.26

*LVEF* left ventricular ejection fraction

^***^p < .001. Italic = trend values (*p* < 0.10); values are *N* (%) for categorical variables and mean (SD) for continuous variables

^a^Test value is Mann-Whitney *U* test for non-parametric continuous cardiac symptoms

A total of 85/256 patients (33%) reported anginal symptoms during CST. However, anginal symptoms were not more common in patients with ischemia (32%) versus patients without ischemia (34%) during CST (see Table 2). When examining the continuous CST-induced symptom scores, the following pattern of results was found: 94 (38%) reported chest pain (mean severity score 2.20 ± 3.13), and 126 (51%) reported dyspnea (mean severity score 2.67 ± 3.11). Other symptoms are reported in Table 2.

### Emotions During CST

Table 3 presents the mean percentages of facial expressions of emotions during CST for the consecutive stress testing time blocks for the full sample. Compared with baseline, sadness, anxiety, and anger showed a significant increase during CST, and happiness decreased. Of the four emotions, sadness (9–12%) was the most frequently expressed emotion during CST. All emotions returned to approximately baseline levels during the recovery phase.

**Table 3:** Facial expressions of emotions for baseline, start CST, maximal CST, and recovery for patients undergoing CST

	Baseline	Start CST			Maximal CST			Recovery		
	Mean ± SD	Mean ± SD	*t* value^a^	Cohen’s d	Mean ± SD	*t* value^a^	Cohen’s d	Mean ± SD	*t* value^a^	Cohen’s d
Negative emotions:										
Sadness	9.0 ± 7.2	11.24 ± 13.27	3.74**	0.24	11.62 ± 12.31	4.04**	0.26	10.13 ± 9.59	2.21*	0.14
Anxiety	2.87 ± 2.64	2.53 ± 2.69	−2.17*	0.14	3.15 ± 3.95	1.15	0.07	3.61 ± 3.27	3.65**	0.23
Anger	2.89 ± 2.89	2.99 ± 3.19	0.51	0.03	3.62 ± 4.08	3.15*	0.20	3.29 ± 3.44	1.89	0.12
Positive emotion:										
Happiness	7.65 ± 7.27	6.60 ± 7.03	−2.72*	0.17	6.87 ± 10.13	1.28	0.08	7.19 ± 7.64	0.95	0.06

*p .05, **p .001

^a^Compared with baseline emotion; *N* = 256

Self-reported anxiety/tension occurred in 17% of the participants during CST (mean severity score 0.71 ± 1.85). The self-reported anxiety/tension response was not correlated with the facial expression of emotions (*τ*_*b*_ < 0.047) indicating that these measures assess different aspects of emotion expression. Female patients more often self-reported anxiety/tension versus male patients (1.00 ± 2.21; 0.49 ± 1.49, *p* = 0.030) during CST, but facial emotion expressions during CST did not differ between males and females for all time blocks (partial eta^2^ = 0.011 for sadness, all *p’s* >  0.215).

We also explored whether patients who participated in the bicycle exercise protocol (*N* = 79) differed from those undergoing CST using adenosine with minimal exertion (*N* = 132) in measured facial emotion expression (see Supplemental Table S1). Levels of happiness were significantly higher in patients receiving exercise-based CST compared with adenosine-based CST for all time blocks compared with baseline (*F*(2.51, 599.10) = 4.82, *p* = 0.005, partial eta^2^ = 0.020). No effects of the type of CST protocol (exercise versus adenosine) were found for the other facial expressions of emotions or self-reported anxiety/tension. The significant increase of negative emotions during CST which was found in the total sample (*N* = 256) was significant only in the adenosine-ischemia group for sadness (Table S1).

### Ischemia and Emotions During CST

**Fig. 1:**
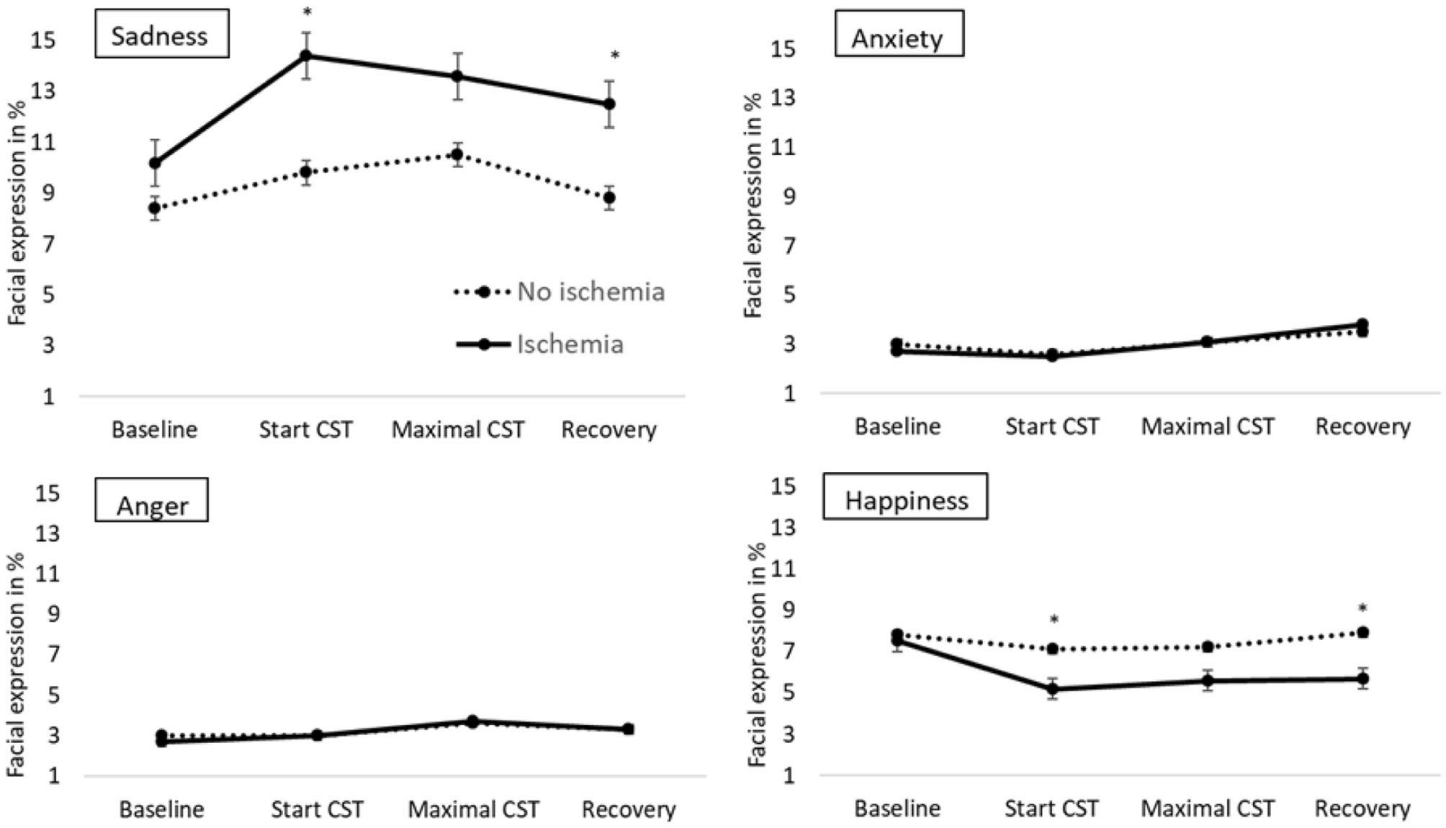
Emotions during CST in patient with versus without ischemia. Data indicate the facial expression of the four emotions (sadness, anxiety, anger, and happiness) at rest, start of CST, maximum CST, and recovery. Error bars indicate standard error of the mean. *p < 0.05

Figure 1 shows the emotional expressions of patients stratified for ischemia (*N* = 89) versus those without ischemia (*N* = 167) during CST. Expressed sadness was higher in patients with ischemia during start CST (14.43 ± 16.05 vs. 9.75 ± 11.18; *t*(129.13) = −2.42, *p* = 0.017, *d* = 0.34), and during recovery (12.49 ± 10.98 vs. 8.83 ± 8.53; *t*(253) = −2.95, *p* = 0.004, *d* = 0.37). Similarly, differences between patients with vs. without ischemia were also found for happiness during start CST (5.16 ± 5.11 vs. 7.14 ± 7.67; *t*(234.41) = 2.44, *p* = 0.015, *d* = 0.30), and happiness during recovery (5.73 ± 6.72 vs. 7.89 ± 7.84; *t*(202.05) = 2.30, *p* = 0.023, *d* = 0.30).

Supplemental Table S2 displays the findings of the repeated measures ANOVA analyses for the change in facial expressions of emotions during CST. There was a significant change in negative emotions over time for facial expression of sadness, anxiety, and anger, but not for happiness (Supplemental Table S2). Patients with versus without ischemia significantly differed in facial expressions of sadness for all time blocks combined (*F*(1, 240) = 6.31, *p* = 0.013, partial eta^2^ = 0.026), but there was no interaction between time block with ischemia status (*F*(2.61, 626.21) = 1.55, *p* = 0.205, partial eta^2^ = 0.006). The effect sizes of the other main effects were < 0.035; see Supplemental Table S2). When adjusting for type of protocol (exercise or adenosine), the main effect for sadness remained significant (*F*(1, 238) = 4.41, *p* = 0.004, partial eta^2^ = 0.018), and the interaction term non-significant (*p* = 0.236, partial eta^2^ = 0.006), and effect sizes for the main effects of the other three emotions also remained non-significant (*p* > 0.087, partial eta^2^ < 0.027) in the models adjusted for protocol type.

The self-reported levels of anxiety/tension during peak CST did not significantly differ between patients with vs. without ischemia (0.40 ± 1.28 vs. 0.87 ± 2.08; *p* = 0.057, *d* = 0.27), although the association was in the expected direction.

### Association of Cardiac Symptoms During CST with Emotions

Supplemental Table S3 presents the correlations between cardiac symptoms with the emotional expressions at the peak levels of CST. Happiness showed a small significant inverse association with chest pain and was positively associated with fatigue. Of the negative emotions (sadness, anxiety, and anger), none showed a significant association with the symptoms during CST.

Findings for anginal symptoms were consistent with the continuous symptom measures, indicating that levels of happiness were significantly lower in patients with anginal symptoms (*τ*_*b*_ = −0.174, *p* = 0.001).

### The Combined Presence of Ischemia and Angina Symptoms as Related to Expression of Emotions

**Fig. 2:**
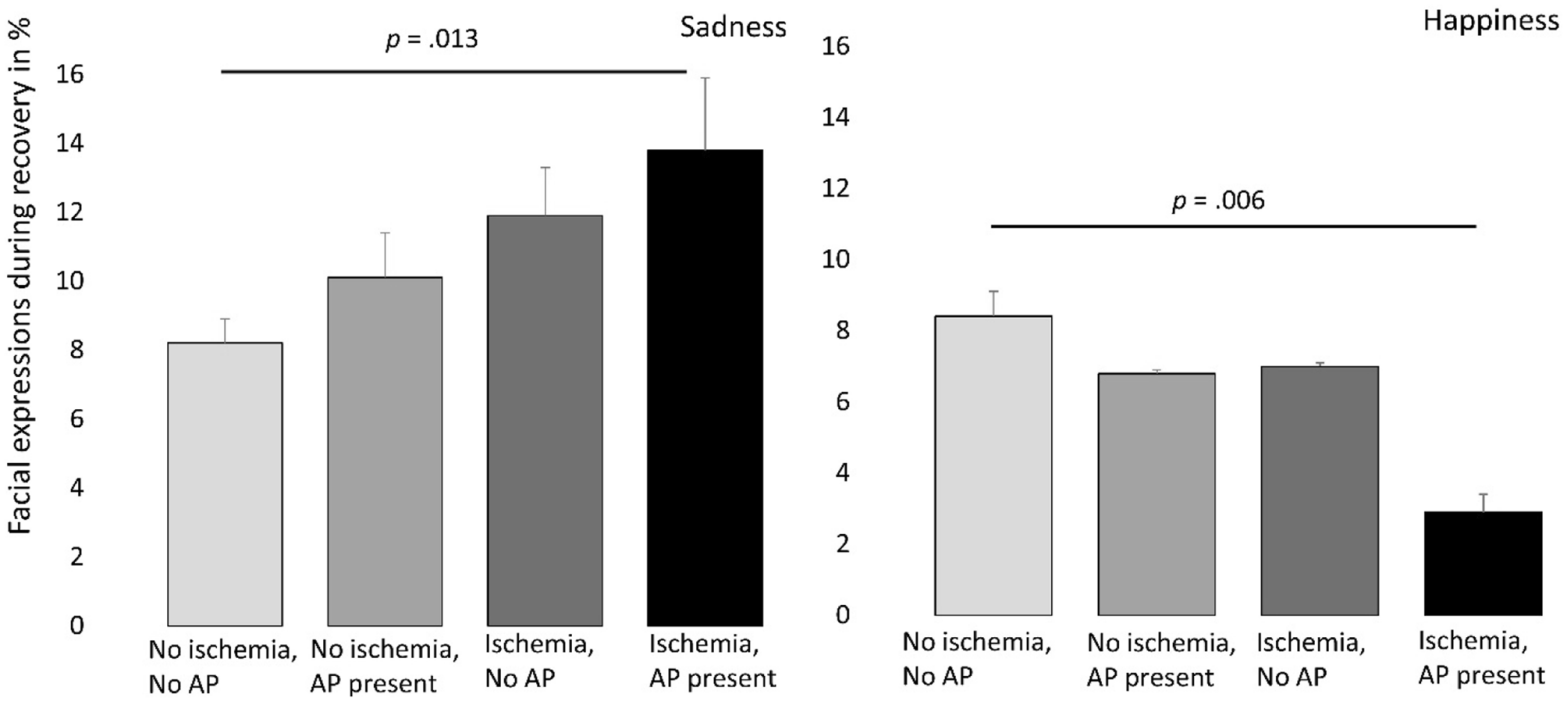
Facial expression of sadness and happiness during recovery of CST for the four ischemia/angina groups. Data are the mean levels of expressed sadness and happiness for four patient groups: (1) without ischemia and no anginal symptoms (reference group: *N* = 110), (2) with cardiac complaints but no ischemia (non-ischemic complaints: *N* = 57), with ischemia but without anginal symptoms (silent ischemia: *N* = 61), and with both ischemia and anginal symptoms (symptomatic ischemia: *N* = 28). Error bars indicate standard error of the mean. A* p* = presence of angina reported during CST

One-way ANOVAs were examined for the facial expressions of emotions between four groups based on the presence/absence of ischemia and occurrence/absence of anginal symptoms during CST. For *baseline*, start of CST, and maximum CST, no significant differences in any of the facial expressions of emotions were found for the four ischemia and angina groups (data not shown). The four groups stratified for ischemia and anginal symptoms and expressed sadness during *recovery* are depicted in Fig. 2. A significant difference was found for sadness during recovery (*F*(3, 254) = 3.67, *p* = 0.013, eta^2^ = 0.042), where sadness was highest in the ischemia-angina group, compared with the reference group with no ischemia and no angina symptoms. During recovery, a significant difference between the groups was also found for happiness (*F*(3, 254) = 4.19, *p* = 0.006, eta^2^ = 0.048), where the ischemia-angina group showed the lowest level of happiness compared with the reference group with no ischemia and no angina symptoms (Fig. 2). We also explored whether there was an interaction between ischemia and anginal symptoms in the emotional responses to CST. The 2 (ischemia) × 2 (anginal symptoms) × 4 (time blocks) analyses revealed main effects for time (*p* < 0.020; eta^2^ ranging from 0.023 to 0.026), but no interaction effects for ischemia × anginal symptoms (all *p* > 0.357, eta^2^ ranging from 0.004 to 0.008).

## Discussion

This study shows that CST resulted in significant increases in facial expressions of sadness, anxiety, anger, and a reduction in happiness compared with baseline measures. Facial expressions of sadness were higher for patients with ischemia versus those without ischemia, which was significant during the start of CST and recovery. In accordance with this finding, facial expressions of happiness were lower for patients with ischemia versus without ischemia during the start of CST and recovery. No differences between patients with versus without ischemia were found for the facial expressions of anxiety and anger. Furthermore, anginal symptoms were associated with emotional expression, particularly a reduction in happiness. More specifically, during recovery, the patients with both ischemia and anginal symptoms showed significantly lower happiness and higher sadness compared with the other three groups. These findings suggest an additive effect of presence of ischemia and anginal symptoms for these facial expressions of emotions during CST. These findings reflecting more expression of sadness and less happiness may be relevant to the assessment of depression and positive affect in patients with ischemic heart disease [2, 4, 38].

The facial expressions of emotions examined in this study changed significantly in response to CST, with analyzed percentages being highest during maximum exertion for negative emotions, except for anxiety, which was highest during recovery. This means that patients overall showed most negative emotions during the most strenuous part of the CST. However, facial expressions of emotions of patients with versus without ischemia only differed for sadness and happiness during the start of CST and recovery and to a lesser extent during maximal CST. One explanation for this pattern of results is that the initial emotional response to CST lowers the threshold for developing ischemia, which would be consistent with the model of mental stress-induced ischemia [11]. Another possibility is that ischemia develops later than the 1-min post tracer injection and that the recovery period used in this study actually coincided with the peak of myocardial ischemia severity. Patients with ischemia also had a more extensive cardiac history (including having undergone more CAGs) and may have a higher ischemic heart disease-related burden than patients without ischemia. Patient expectations prior to CST (based on the frequency and severity of cardiac symptoms or uncertainty about the diagnosis) may have influenced the emotional state during CST. We took “baseline” emotion expression prior to start of CST into consideration, and the data suggest that prior experiences may indeed affect the expression of sadness prior to CST (Fig. 1). The data also indicate, however, that the observed “effects” of ischemia and anginal chest pain were not the result of these baseline differences. Uncertainty about the disease and current severity and frequency of anginal symptoms may be effect-modifying factors and require further research. The higher facial expressions of sadness and lower happiness shown by patients with ischemia during CST could therefore reflect illness-related disease burden in general rather than the sequelae of myocardial ischemia per se. The present data indicate, however, that ischemia and also anginal symptoms resulted in a (short-term) increase in negative emotions, particularly sadness and reduced happiness in patients referred for diagnostic testing for inducibility of myocardial ischemia.

In the current study sample, patients with ischemia did not experience more anginal symptoms or chest pain complaints during CST than patients without ischemia. The dissociation between ischemia and cardiac symptoms is well documented in both daily life settings (e.g., silent ischemia during Holter monitoring) as well as induced ischemia during CST [39]. This finding could be related to research linking psychological distress and negative emotions to silent ischemia [27]. In the additional ischemia and angina group analyses, we did not find results indicating that the ischemia-no angina group showed more facial expressions of negative emotions than the other groups. However, the increase in facial expression of sadness in response to silent ischemia during cardiac stress testing might be a response to the autonomic nervous system withdrawal that typically accompanies myocardial ischemia, but it is also possible that patients experienced discomfort that they did not report but that nonetheless could be detected from facial expression of negative affect such as increased sadness and reduced happiness. Nonetheless, it would be interesting to take other psychosocial factors into account when comparing these groups, especially considering the important role of depression in chest pain [38]. Of the emotions analyzed in this study, only happiness was inversely associated with anginal symptoms and chest pain during CST. This finding is partly consistent with the literature. Prior research has demonstrated associations between negative emotions, such as sadness and anger, and cardiac symptoms [16, 20, 25, 40]. The present study found stronger effects for reduced facial expression of happiness as related to chest pain rather than negative emotions such as sadness, anxiety, or anger, which is not in line with the prior studies. This study analyzed facial expressions during CST in a hospital setting which is a different environmental context than what has been investigated in most previous studies. The hospital setting could have had an attenuating effect on someone’s expressiveness, both verbally and non-verbally [41]. However, the findings related to happiness may be of interest not only in the context of the health advantages associated with personality traits and other psychological factors associated with high levels of positive affect [5, 6] but also because of the possibility that expressions of positive emotions (e.g., smiling) may result in a reduced likelihood for inducibility of ischemia during CST. However, this notion requires empirical validation and the main point of this article is to document the reverse pathway, that is, the effects of ischemia on expression of positive and negative emotions.

We observed that patients undergoing pharmacological CST showed less happy facial expressions than patients undergoing bicycle exercise CST. Partly, these findings were to be expected, since patients undergoing CST with adenosine tend to experience more symptoms overall compared with CST using bicycle exercise [36, 42]. In this context, it is interesting that only happiness, but no other analyzed emotion, was different for patients performing adenosine vs. bicycle exercise CST.

The present findings should be evaluated in light of a number of study limitations. The patient sample of the present study is rather heterogeneous. Patients were categorized into presence or absence of ischemia, but underlying significant coronary obstructions and cardiac history (other than previous myocardial infarction, CAG, PCI, or CABG) were not taken into account in forming subgroups. More extensive cardiac history should be taken into account in future research. Furthermore, some patients have a lower or higher pain threshold than others, possibly resulting in more or less symptom reporting respectively [43]. Gender differences were not specifically taken into account in the statistical analyses as it was not the main focus of the present study, but the impact of gender differences on cardiac-related outcomes has been previously documented. For example, atypical chest pain is more common in women than men with ischemic heart disease (IHD) and chest pain in women is less likely to be related to obstructive coronary artery disease (CAD) than in men [44–46]. These differences in clinical presentation can lead to less diagnostic testing for inducible ischemia in women, as well as delays in diagnosis compared with men [47, 48]. Furthermore, the prevalence of psychological factors in cardiac patients, such as anxiety, is higher in women than in men [49], and this higher prevalence has been associated with the presence of inducible ischemia [50]. However, other previous findings of the THIO study indicated no significant differences in ischemia between men and women with (both state and trait) anxiety [51]. For future research, gender differences in emotional expression during CST should be investigated with a larger sample and with more attention for underlying cardiac history, so that different groups (CAD vs. non-obstructive CAD, for example) in relation to gender can be investigated.

Although the assessment of symptoms during CST was conducted in a standardized manner, some patients needed to be probed more than others to indicate experienced symptoms during CST on a scale from 1 to 10. Some patients had difficulty with translating their experienced symptoms into a scaled number, possibly more so during CST than in a quiet research setting. In addition to digitally analyzed facial expressions of emotions, the self-reported emotion measure was limited to a question about anxiety/tension patients experienced during CST. Other emotion-related questions concerning emotional states (e.g., anger) seemed out of place during CST, as the protocol is a physical challenge procedure and patients are not likely to self-report emotions such as anger. However, the emotional state prior to CST other than anxiety/tension may be important to evaluate in future research. The findings of this study require replication in larger samples with more elaborate adjustment for potentially confounding and effect-modifying factors and adjustment of the familywise error related to multiple statistical testing.

In conclusion, patients with ischemia displayed higher levels of sadness and lower levels of happiness than patients without ischemia when using facial expression data, indicating that the valence aspect of emotions (sadness and reduced happiness) may be more important than emotion expression with a high arousal component (i.e., anxiety and anger) in response to CST. A similar pattern was found for anginal symptoms, particularly reduced expressed happiness. Although the present findings took baseline emotional states into account, more work is needed to clarify patients’ daily experiences with cardiac complaints and their perceived uncertainty about the diagnostic procedure into account when evaluating emotional states during CST. Nonetheless, if myocardial ischemia produces an increase in negative emotions and a decrease in happiness, as suggested by the present data, these responses to ischemia may increase the risk of depressive symptoms and reduce positive affect in patients with ischemic heart disease. Future studies are also needed to compare different patient subgroups, for example, those with versus without obstructive coronary artery disease and/or coronary microvascular disease, to shed a broader light on the heterogeneous group of patients referred for diagnostic testing for ischemic heart disease. The measurement of facial expressions of emotions is relatively new, and this study provides the first steps of incorporating these measurements in research on emotions and reported cardiac symptoms in patients with ischemic heart disease.

- Informed consent was obtained from all individual participants included in the study.
- All procedures performed in studies involving human participants were in accordance with the ethical standards of the institutional and/or national research committee and with the 1964 Helsinki declaration and its later amendments or comparable ethical standards.

## Supplementary Information

Below is the link to the electronic supplementary material.

Supplementary file1 Figure S1 Flow chart of procedures and CST protocol. The myocardial perfusion imaging was conducted using a 2-day assessment protocol. The rest day protocol involved resting imaging at which informed consent for the project was obtained. The Stress day protocol involved imaging following CST during which video recordings were made for digital analysis of facial expressions of emotions (DOCX 32 KB)

Supplementary file2 (DOCX 17 KB)

Supplementary file3 (DOCX 14 KB)

Supplementary file4 (DOCX 13 KB)

## Abbreviations

CST: Cardiac stress testing
THIO: The Heart Inside Out study
BMI: Body mass index
LVEF: Left ventricular ejection fraction
CAG: Coronary angiography
