# Supplementary material for: Facial Expressions of Emotions During Pharmacological and Exercise Stress Testing: the Role of Myocardial Ischemia and Cardiac Symptoms
Source: Int J Behav Med. 2021 Feb 23;28(6):692–704. doi: 10.1007/s12529-021-09963-3 (PMC8551126; doi:10.1007/s12529-021-09963-3)
Supplement: Supplementary file 1 — Supplementary file1 Figure S1 Flow chart of procedures and CST protocol. The myocardial perfusion imaging was conducted using a 2-day assessment protocol. The rest day protocol involved resting imaging at which informed consent for the project was obtained. The Stress day protocol involved imaging following CST during which video recordings were made for digital analysis of facial expressions of emotions (DOCX 32 KB) [file 12529_2021_9963_MOESM1_ESM.docx]

Figure S1 Flow chart of procedures and cardiac stress-testing protocol.

1-3 days interval

Video recordings and report of cardiac and anxious/ tense symptoms and intensity

**Info & informed consent,**

**Fill out questionnaire**

**Email info on study**

Rest day protocol

Tc-99m injection

WR 45 min

MPI 20 min

Days before

**Recovery**: 1 min slow down

**Max CST**: 1 min peak adenosine or at target HR Tc99M injection

**Start CST**: 2 min adenosine or 3-10 min cycling

Stress day protocol

WR

CST protocol ±15 min

**Baseline:**

Info on recording, Start video recording ±1 min

**Questionnaire (or take home)**

WR 45 min

MPI 20 min
