# Supplementary material for: Facial Expressions of Emotions During Pharmacological and Exercise Stress Testing: the Role of Myocardial Ischemia and Cardiac Symptoms
Source: Int J Behav Med. 2021 Feb 23;28(6):692–704. doi: 10.1007/s12529-021-09963-3 (PMC8551126; doi:10.1007/s12529-021-09963-3)
Supplement: Supplementary file 2 — Supplementary file2 (DOCX 17 KB) [file 12529_2021_9963_MOESM2_ESM.docx]

Supplemental Table S1. Facial expressions of emotions for baseline, start CST, maximal CST, and recovery for patients undergoing CST stratified for protocol and ischemia presence or absence.

|  | Baseline | Start CST | | | | | | Maximal CST | | | | Recovery | | |  |
| --- | --- | --- | --- | --- | --- | --- | --- | --- | --- | --- | --- | --- | --- | --- | --- |
| **Exercise-No ischemia** | Mean ± SD | Mean ± SD | | t-value^a^ | | Cohen’s d | | Mean ± SD | | t-value^a^ | Cohen’s d | Mean ± SD | t-value^a^ | Cohen’s d | |
| Sadness | 8.43±6.26 | 10.40±13.37 | | 1.39 | | 0.19 | | 10.76±13.60 | | 1.48 | 0.22 | 8.12±8.43 | -0.33 | 0.04 | |
| Anxiety | 3.09±2.61 | 2.55±2.74 | | *-1.71* | | 0.20 | | 3.31±4.69 | | 0.36 | 0.06 | 4.20±3.14 | **3.23*** | 0.38 | |
| Anger | 2.75±2.94 | 3.28±3.63 | | 1.07 | | 0.16 | | 3.66±4.24 | | 1.73 | 0.25 | 3.08±2.55 | 0.84 | 0.12 | |
| Happiness | 8.88±7.44 | 9.18±8.40 | | 0.30 | | 0.04 | | 11.42±14.36 | | 1.45 | 0.22 | 10.57±8.51 | 1.50 | 0.21 | |
| **Exercise-Ischemia** |  |  | |  | |  | |  | |  |  |  |  |  | |
| Sadness | 8.49±5.88 | 12.80±14.05 | | 1.62 | | 0.85 | | 11.75±10.60 | | 1.27 | 0.38 | 8.11±6.85 | -0.43 | 0.06 | |
| Anxiety | 2.75±1.54 | 1.77±1.13 | | **-3.24*** | | 0.73 | | 3.21±5.17 | | 0.42 | 0.12 | 2.63±1.59 | 1.46 | 0.08 | |
| Anger | 3.06±2.78 | 3.71±4.21 | | 0.77 | | 0.18 | | 3.34±3.59 | | 0.36 | 0.09 | 3.47±3.50 | 0.64 | 0.13 | |
| Happiness | 11.52±9.80 | 7.44±5.79 | | **-2.72*** | | 0.51 | | 10.80±15.06 | | -0.21 | 0.06 | 9.31±9.60 | -0.83 | 0.23 | |
| **Adenosine-No ischemia** |  |  | |  | |  | |  | |  |  |  |  |  | |
| Sadness | 8.48±6.65 | 9.54±10.43 | | 1.26 | | 0.12 | | 10.07±10.25 | | 1.58 | 0.18 | 8.81±7.87 | 0.45 | 0.05 | |
| Anxiety | 2.73±2.53 | 2.38±2.35 | | -1.26 | | 0.14 | | 3.20±4.16 | | 1.07 | 0.14 | 3.06±2.94 | 1.09 | 0.12 | |
| Anger | 3.24±3.11 | 2.93±2.97 | | -0.94 | | 0.10 | | 3.61±4.42 | | 0.88 | 0.10 | 3.16±3.10 | -0.24 | 0.03 | |
| Happiness | 6.64±5.12 | 5.98±5.87 | | -1.04 | | 0.12 | | 4.73±5.40 | | **-2.82*** | 0.36 | 6.56±6.29 | -0.13 | 0.01 | |
|  | Baseline | Start CST | |  | |  | | Maximal CST | |  |  | Recovery |  |  | |
| **Adenosine-Ischemia** | Mean ± SD | Mean ± SD | | t-value^a^ | | Cohen’s d | | Mean ± SD | | t-value^a^ | Cohen’s d | Mean ± SD | t-value^a^ | Cohen’s d | |
| Sadness | 11.06±9.77 | 15.26±17.80 | | **2.81*** | | 0.29 | | 15.30±15.27 | | **2.94*** | 0.33 | 13.14±12.22 | **2.12*** | 0.19 | |
| Anxiety | 3.07±2.44 | 2.82±2.69 | | -0.85 | | 0.10 | | 3.27±2.97 | | 0.54 | 0.07 | 4.07±3.41 | **2.30*** | 0.34 | |
| Anger | 2.15±2.32 | 2.34±2.25 | | 0.67 | | 0.08 | | 3.47±3.37 | | **2.68*** | 0.46 | 2.81±2.88 | 1.52 | 0.25 | |
| Happiness | 6.28±5.66 | 4.92±5.50 | | -1.60 | | 0.24 | | 4.80±7.50 | | -1.29 | 0.22 | 4.57±4.37 | *-1.80* | 0.34 | |
| **Adenosine-Lying down-No Ischemia** | | |  | |  | |  | |  |  |  |  |  |  | |
| Sadness | 8.32±4.52 | 9.21±9.02 | | 0.53 | | 0.12 | | 12.09±12.33 | | 1.65 | 0.41 | 11.66±10.89 | 1.76 | 0.40 | |
| Anxiety | 3.37±4.30 | 3.24±4.04 | | -0.18 | | 0.03 | | 2.66±2.56 | | -1.06 | 0.20 | 3.60±4.08 | 0.21 | 0.05 | |
| Anger | 2.56±1.89 | 2.80±2.87 | | 0.50 | | 0.10 | | 3.65±4.25 | | 1.50 | 0.33 | 4.47±5.83 | 1.83 | 0.44 | |
| Happiness | 9.53±12.35 | 7.44±10.65 | | **-2.14*** | | 0.18 | | 7.42±10.80 | | -1.05 | 0.18 | 6.87±10.07 | -1.53 | 0.24 | |
| **Adenosine-Lying down-Ischemia** | | |  | |  | |  | |  |  |  |  |  |  | |
| Sadness | 9.86±8.74 | 12.88±15.10 | | 1.48 | | 0.24 | | 11.51±10.11 | | 1.02 | 0.17 | 14.93±10.90 | 1.83 | 0.51 | |
| Anxiety | 1.82±1.33 | 2.32±2.88 | | 0.95 | | 0.22 | | 2.77±3.12 | | 1.49 | 0.40 | 2.96±2.83 | 1.93 | 0.52 | |
| Anger | 3.81±3.90 | 3.47±4.0 | | -0.72 | | 0.09 | | 4.14±4.36 | | 0.56 | 0.08 | 3.83±4.0 | 0.03 | 0.005 | |
| Happiness | 5.31±3.68 | 3.90±2.88 | | *-1.83* | | 0.43 | | 3.44±2.90 | | **-2.25*** | 0.56 | 4.63±7.47 | -0.35 | 0.12 | |
| * p < .05, ** p < .001;  ^a^ compared to baseline emotion; Italic = trend values (p < 0.10); N = 256. | | | | | | | | | | | | | | | |
