# Supplementary material for: Facial Expressions of Emotions During Pharmacological and Exercise Stress Testing: the Role of Myocardial Ischemia and Cardiac Symptoms
Source: Int J Behav Med. 2021 Feb 23;28(6):692–704. doi: 10.1007/s12529-021-09963-3 (PMC8551126; doi:10.1007/s12529-021-09963-3)
Supplement: Supplementary file 3 — Supplementary file3 (DOCX 14 KB) [file 12529_2021_9963_MOESM3_ESM.docx]

Table S2. Repeated measures ANOVA of facial expressions of emotions during cardiac stress-testing.

|  | **Negative emotions** | | | | | |
| --- | --- | --- | --- | --- | --- | --- |
| Emotion | | F-value | d.f. | Sig. | Partial eta^2^ | |
| **Sadness** | |  |  |  |  | |
| time | | 8.81 | 2.61, 626.21 | **<.001** | .035 | |
| ischemia | | 6.31 | 1, 240 | **.013** | .026 | |
| time*ischemia | | 1.55 | 2.61, 626.21 | .205 | .006 | |
| **Anxiety** | |  |  |  |  | |
| time | | 9.87 | 2.62, 627.91 | **<.001** | .040 | |
| ischemia | | .005 | 1,240 | .946 | <.001 | |
| time*ischemia | | 0.44 | 2.62, 627.91 | .699 | .002 | |
| **Anger** | |  |  |  |  | |
| time | | 4.81 | 2.75, 660.69 | **.003** | .020 | |
| ischemia | | .113 | 1, 240 | .737 | <.001 | |
| time*ischemia | | 0.07 | 2.75, 660.69 | .968 | <.001 | |
| **Positive emotion** | |  |  |  |  | |
| **Happiness** | |  |  |  |  | |
| time | | 2.20 | 2.48, 595.70 | *.099* | .009 | |
| ischemia | | 2.85 | 1, 240 | *.093* | .012 | |
| time*ischemia | | 0.96 | 2.48, 595.70 | .399 | .004 | |
| Bold = group differences significant at p < .05 level; Italic = trend values (p < 0.10) Mauchly’s test of sphericity. indicated that the assumption of sphericity had been violated (χ^2^(5) = 68.218, *p* < .001. A Greenhouse-Geisser correction was used to calculate the results of the repeated measures ANOVA | | | | | |  |
