# Supplementary material for: Facial Expressions of Emotions During Pharmacological and Exercise Stress Testing: the Role of Myocardial Ischemia and Cardiac Symptoms
Source: Int J Behav Med. 2021 Feb 23;28(6):692–704. doi: 10.1007/s12529-021-09963-3 (PMC8551126; doi:10.1007/s12529-021-09963-3)
Supplement: Supplementary file 4 — Supplementary file4 (DOCX 13 KB) [file 12529_2021_9963_MOESM4_ESM.docx]

Supplemental Table S3. Correlations of facial expressions during maximum exertion and cardiac symptoms during cardiac stress-testing.

|  | Negative emotions | | | Positive emotions |
| --- | --- | --- | --- | --- |
|  | Sadness | Anxiety | Anger | Happiness |
| Anginal symptoms | .015 | .080 | -.048 | **-.174*** |
| Cardiac symptoms reported | | | | |
| Chest pain | .018 | .042 | -.043 | **-.161*** |
| Shortness of breath | -.032 | .089 | -.036 | .013 |
| Dizziness | .019 | -.074 | -.035 | -.020 |
| Nausea | .051 | -.022 | -.032 | -.042 |
| Fatigue | .001 | .058 | .027 | **.131*** |
| Flushing | -.007 | .030 | -.077 | -.005 |
| Other^a^ | .041 | .027 | -.001 | -.054 |
| *p<.01; ^a^ Other symptoms consist of for example headaches, dry throat, muscle tension or muscle spasms | | | | |
